# Cardiac magnetic resonance imaging (cMRI) as a first-line approach to subclinical heart failure in children undergoing cardiotoxic chemotherapy

**DOI:** 10.1186/1532-429X-13-S1-P177

**Published:** 2011-02-02

**Authors:** Maëlle de Ville de Goyet, Benedicte Brichard, Thierry Pirotte, Stephane Moniotte

**Affiliations:** 1 Cliniques Saint Luc, UCL, Brussels, Belgium

## Background

Cardiotoxic chemotherapy including anthracyclines remains a standard of care in paediatric cancers. Recent data suggest that heart failure secondary to chemotherapy is, to some degree, reversible, but requires early detection and prompt intervention. Since a substantial proportion of paediatric patients still develop heart failure, this work aims to develop a follow-up protocol based on cMRI to evaluate the patient’s cardiovascular function and haemodynamics, as well as myocardial iron overload, perfusion and viability.

## Material and methods

From October 2009 to September 2010, 38 children (mean age 7,8 years (0,3-17,6); mean BSA of 0,98m^2^) were prospectively identified as candidates for cardiotoxic chemotherapy. All patients underwent a cMRI to evaluate 1) systolic and diastolic function on steady-state free precession cine and tagged imaging, 2) myocardial perfusion and viability after Gadolinium injections, 3) mitral and aortic flows through velocity-encoded sequences, and 4) myocardial and liver iron content based on T2* MRI.

## Results

The study protocol was completed in all patients within the first month of diagnosis, under general anaesthesia for the 14 youngest patients. The mean MRI acquisition time 40+/-9min. Systolic biventricular volumes, mass and function as well as left ventricular (LV) diastolic function were normal in all patients, with a mean LV EF of 62,2+/-8% and RV EF of 49,5+/-7,2%. Although a perfusion defect was not retrieved in any of these pre-chemotherapy assessments, mild LV myocardial scaring (mean of 4,5%) was frequently identified by gadolinium-enhanced imaging. Aortic and mitral flux showed normal LV stroke volumes (mean of 3,78L/min/m^2^). No significant liver or cardiac iron overload was detected (mean T2* of 23,6 and 33,2msec, respectively). Evaluation of the inter-observer and intra-observer variability was performed for all variables studied and confirmed that the coefficients of determination were very strong (R^2^>0,95), as previously reported by others.

## Conclusions

Our preliminary study shows that a detailed cardiovascular evaluation of young patients undergoing chemotherapy is achievable in a reasonable time in the early phase following diagnosis. We believe that the low intra and inter-observer variability of cMRI makes it suitable for a complete mid- and/or long-term follow-up of cardiovascular function. This should allow to evaluate the true harmful effects of cardiotoxic medications used against paediatric cancers and to confirm the potential role of cMRI as a first-line approach to subclinical heart failure in children.

